# The impact of vitamin D supplement intake on vascular endothelial function; a systematic review and meta-analysis of randomized controlled trials

**DOI:** 10.1080/16546628.2016.1273574

**Published:** 2017-03-20

**Authors:** Mohsen Mazidi, Ehsan Karimi, Peyman Rezaie, Hassan Vatanparast

**Affiliations:** ^a^ Key State Laboratory of Molecular Developmental Biology, Institute of Genetics and Developmental Biology, Chinese Academy of Sciences, Beijing, China; ^b^ Institute of Genetics and Developmental Biology, International College, University of Chinese Academy of Science (IC-UCAS), Chaoyang, China; ^c^ Biochemistry and Nutrition Research Center, School of Medicine, Mashhad University of Medical Science, Mashhad, Iran; ^d^ College of Pharmacy and Nutrition, University of Saskatchewan, Saskatoon, Canada

**Keywords:** Meta-analysis, vitamin D, endothelial function, flow-mediated dilation

## Abstract

**Aim:** to systematically review and conduct a meta-analysis of randomized controlled trials investigating the impact of vitamin D supplementation on endothelial function.

**Method:** We searched PubMed-Medline, SCOPUS, Web of Science and Google Scholar (until June 2016) to detect prospective studies evaluating the impact of vitamin D supplementation on endothelial function indexes. We used random effects models (using DerSimonian-Laird method) and generic inverse variance methods to synthesize quantitative data. We used the leave-one-out method for sensitivity analysis. To quantitatively assess the heterogeneity we used the I^2^ index. Systematic review registration: CRD42016039329.

**Results:** From a total of 213 entries identified, 12 studies were appropriate for inclusion into the final analysis. The meta-analysis indicated a significant enhancement in flow-mediated dilation (FMD) following D supplementation (vitamin D intervention group versus control group 1.27 %, (95% CI 0.20 to 2.34, N = 11 arms, heterogeneity p = 0.054; I^2^ 51.2 %). These findings were robust in sensitivity analyses.

**Conclusions:** This meta-analysis suggested that vitamin D supplementation may improve endothelial function. Randomized control trials with a longer-term follow-up are warranted to clarify the existing controversies and shed light on the potential underlying mechanisms.

## Introduction

The significant role of vitamin D in bone health and calcium homeostasis is well-documented [1]. However, emerging evidence indicates that vitamin D has important functions on other body systems including the cardiovascular system [2]. Recent observational studies have reported an association between vitamin D deficiency and hypertension [3], incident cardiovascular disease (CVD) [4, 5], myocardial infarction [6], cardiovascular death [7] and total mortality [8]. Clinical studies have revealed that vitamin D supplement consumption improves endothelium-dependent vasodilation, a predictor of cardiovascular issues[9, 10], among patients with diabetes [11] and as well as healthy adults with vitamin D deficiency [12]. The vascular endothelium has a pivotal role in responding to blood-borne signals and alterations in haemodynamic forces. The future development of CVD [13] and the prediction of type 2 diabetes (T2DM) are strongly associated with endothelial dysfunction [13,14]. Vitamin D has recently been proposed to have potential cardioprotective properties particularly through its actions on the endothelium [13]. However, the putative mechanisms of action of vitamin D through which it may effect on the atherosclerotic process have not been completely elucidated [13]. This may in part be through augmented nitric oxide (NO) production, reduced oxidative stress, decreased expression of interleukin 6 (IL-6) expression, or vascular cell adhesion molecules (VCAM) and intracellular adhesion molecule (ICAM) [15]. It has been shown that the vascular expression of NF-κB was higher in patients with vitamin D deficiency versus vitamin D-sufficient patients and that the endothelial expression of the downstream pro-inflammatory cytokine IL-6 was higher in deficient in comparison with sufficient subjects [16]. The vitamin D receptor (VDR) expression and 1-alpha-hydroxylase were also decreased in vitamin D-deficient patients which could be one of the molecular mechanism explaining the effects [16]. Vitamin D supplementation has been recognized to regulate the levels of inflammatory cytokines, including TNF-α and IL-6 in addition to preventing lipopolysaccharide (LPS) induced activation and vasodilatation of vascular endothelium in vitro [17]. Hence, the effects of vitamin D on the vascular system could be mediated by its effects on the inflammatory process that causes an augmented endothelial expression of nuclear factor-κB (NF-κB), increased concentrations of downstream product IL-6 [16], VCAM and ICAM induced by tumour necrosis factor (TNF)-induced [18]. The potential effect of vitamin D supplement consupmtion on endothelial function is not well-understood. Single studies to date have been limited by sample size, research design and subject traits (gender, ethnicity, age, etc.), and generally underpowered to achieve a comprehensive and reliable conclusion. To overcome such limitations, a meta-analysis which pools data from existing studies can be used. Hence, we decided to conduct a meta-analysis in order to clarify the potential impact of vitamin D supplementation on endothelial function by systematically reviewing the existing randomized control trials and available meta-analysis data.

## Materials and methods

### Strategy of literature search

We conducted this study following the Preferred Reporting Items for Systematic Reviews and Meta-Analyses (PRISMA) Guidelines [19,20]. We registered our study protocol with the International Prospective Register of Systematic Reviews, PROSPERO (registration no: CRD42016039329). Our primary exposure of interest was vitamin D supplement consumption, while the main outcome of interest was the changes in the endothelial function indexes subsequent to vitamin D supplementation. We searched multiple databases including Cochrane Database of Systematic Reviews (CDSR), Web of Science and MEDLINE, PUBMED/Medline, as well as Cochrane Central Register of Controlled Trials (CCTR), until June 2016 using a combination of search term available in Supplementary Table 1. As presented in Table 1, we searched for broader endothelial function indexes such as intima-media thickness, nitrate-mediated dilation, flow-mediated dilation; however, because of the lack of data, we have focused mostly on flow-mediated dilation in this study. We only included randomized control trials in this systematic review. We used the wild-card term ‘*’ to enhance the sensitivity of the search strategy. We included published studies in all languages. We hand searched the reference list of qualified articles and conducted email correspondences with authors for additional data where relevant.

**Table 1.** General characteristic of the included studies.

Author, references, year of publication	Country	Study design	Status	Sample size	Sex (% of women)	Mean age	Supplemented dose of vitamin D (IU/day)	Follow-up duration	Vitamin D status
Gepner A, 2012[21]	USA	A prospective, randomized, double-blind, placebo controllled trial	post-menopausal women with serum 25(OH)D concentrations between 10 and 60 ng/mL,	114	100%	Test: 63.6 Control: 64.1	2500 IU of oral D3/day	4 months	Baseline:96.35 nmol/l Changes during follow-up: 49.92 nmol/l
Harris A, 2011[22]	USA	A double-blind, randomized, placebo controlled clinical trial	Overweight subjects between the ages of 19 and 50	Test: 22 Control: 23	Test: 59% Control: 48%	Test: 29 ± 2 Control: 31 ± 2	60,000 IU monthly supplementation of oral vitamin D3	16 weeks	Baseline:34.3 nmol/l Changes during follow-up: 75.46 nmol/l
Longenecker C, 2012[23]	USA	A randomized, double-blind, placebo-controlled trial	HIV-infected adults on stable antiretroviral therapy (ART) with durable virological suppression and a baseline 25(OH)D level .20 ng/ml.	Test: 30 Control: 15	Test: 17% Control: 33%	Test: 47 ± 8 Control: 40 ± 10	4,000 IU daily D3	12 weeks	Baseline:28.60 nmol/l Changes during follow-up: 15.9 nmol/l
Sokol S, 2012[24]	USA	a randomized, double-blind, placebo-controlled trial.	Patients with coronary artery disease	Test: 45 Control: 45	Test: 20% Control:33%	Test: 55 ± 9.6 Control: 56.96 ± 11.6	50,000 IU of oral ergocalciferol weekly	12 weeks	Baseline (median):41.34 nmol/l Endpoint (median): 127.2 nmol/l
Sugden A, 2007[11]	UK	Double-blind, parallel group, placebo-controlled randomized trial	Patients with Type 2 diabetes	Test: 17 Control: 17	Test: 41% Control: 53%	Test: 64.9 ± 10.3 Control: 63.5 ± 9.5	Single dose of 100 000 IU vitamin D2	8 weeks	Baseline:40.2 nmol/l Changes during follow-up: 22.9 nmol/l
Witham M, 2013[25]	UK	A randomized, placebo controlled, parallel group, double blinded study	Healthy South Asian women with baseline serum 25-hydroxyvitamin D levels of <75 nmol/L	Test: 25 Control: 25	100%	Test:41.7 Control: 39.4	a single dose of 100,000 units of oral vitamin D3	8 weeks	Baseline:27 nmol/l Changes during follow-up: 10 nmol/l
Witham M, 2013[26]	UK	A double-blind, parallel group, placebo-controlled randomized trial	Had a history of myocardial infarction	Test: 39 Control:39	Test: 28% Control: 33%	Test: 64.3 Control: 67.5	100,000 units of oral vitamin D3	4 months	Baseline:49 nmol/l Changes during follow-up: 13 nmol/l
Witham M, 2015[27]	UK	Parallel-group, double-blind, randomized placebo-controlled trial	Patients with chronic fatigue syndrome	Test: 25 Control: 25	Test: 72% Control: 80	Test: 48.1 Control: 50.7	100,000 units oral vitamin D3	6 months	Baseline:44 nmol/l Endpoint: 64 nmol/l
Witham M, 2010[28]	UK	This was a randomized, parallel group, placebo controlled trial.	Patients with type 2 diabetes and baseline 25-hydroxyvitamin D levels <100	Test: 39 Control: 20	Test: 16% Control: 45%	Test: 65.3 Control: 66.7	(100,000 IU or 200,000 IU) D3	16 weeks	Baseline:41 nmol/l Endpoint: 63 nmol/l
Witham M, 2012[29]	UK	Randomized, placebo-controlled, double-blind trial	Patients with a history of stroke and baseline 25-hydroxyvitamin D levels <75 nmol/L	Test: 30 Control: 28	Test:40% Control: 14%	Test: 66.2 Control: 67.7	100,000 units of oral vitamin D2	16 weeks	Baseline:38.7 nmol/l Endpoint: 54 nmol/l
Yiu Y, 2013[30]	Hong Kong	Double-blind, placebo-controlled trial	Patients with type 2 DM who had suboptimal vitamin D status	Test: 50 Control: 50	Test: 46% Control: 54%	Test: 65.8 Control: 64.9	5000 IU/day D3	12 weeks	Baseline:67.09 nmol/l Endpoint: 186.3 nmol/l
Zoccali C, 2014[31]	Italy	Double-blind, randomized, parallel group trial	All patients with stage 3 to 4 chronic kidney disease	Test: 44 Control: 44	Test: 41% Control: 30%	Test: 63 Control:62	2 µg paricalcitol daily	12 weeks	Baseline:33 nmol/l Changes during follow-up: 5.1 nmol/l

### Selection criteria

**Figure 1.**
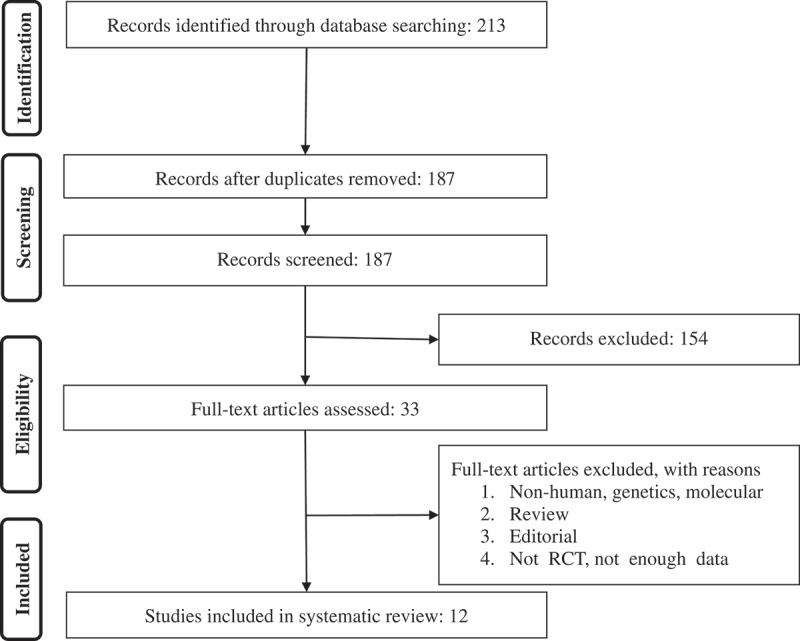
PRISMA flow chart for the studies selection.

All prospective studies that evaluated the association between vitamin D supplementation and the outcome of interest were collected. The inclusion criteria for studies were 1) controlled trials with either crossover or parallel design, 2) prospective studies of patients treated with vitamin D supplement in comparison to control group (either no vitamin D supplement or placebo), 3) demonstration of satisfactory information on primary outcome at that baseline and at the end of follow-up in each group; alternatively providing the net change values. We considered the following exclusion criteria: (i) non-clinical studies; (ii) observational studies with cross-sectional, case-control or cohort design w; and (iii) studies that missed presenting mean (or median) of the main outcome of our interest at baseline and/or the end of the trial. Narrative reviews, commentaries, opinion pieces, methodological papers, editorials, letters as well as publications missing primary data and/or clear description of the methods were also excluded. Study selection started with the removal of duplicates; followed by titles and abstracts screening by two reviewers. To avoid bias, they were blinded to the names, qualifications or the institutional affiliations of the study authors. The agreement between the reviewers was excellent (Kappa index: 0.89; p < 0.001). We resolved the potential disagreements among reviewers before retrieving the selected articles (a flow chart is available in Figure 1).

### Data extraction and management

We retrieved the full text of studies that met the inclusion criteria. Further, two of the reviewers (MM, EK) screened them to cross-check eligibility. After evaluating the quality of methodological approach, the two reviewers (MM, EK) independently summarized the most important information from each study and entered the information into a pre-designed data extraction form. After comparing the independent summaries, the third reviewer (PR) were consulted to resolve the potential differences of opinion. The first reviewer conducted additional necessary further calculations on study data. This step was followed by cross-checking through the second reviewer. Descriptive data that were extracted included the first author, year, country, design, inclusion criteria, age range, total sample size, gender, dose (IU) vitamin D supplementation and follow-up durations (week) were summarized in Table 1. An independent reviewer confirmed all data entries.

## Quality assessment

We used the Cochrane criteria to systematically assess bias in the eligible RCTs [32]. We used the following items for evaluating each study: the soundness of random sequence generation, distribution concealment, blinding of participants in groups, evaluation of-of personnel, and outcome, management of drop-outs (data with the incomplete outcome), discerning in reporting the outcome, as well as any other potential bias. A judgement of ‘yes’ designated low risk of bias, while ‘no’ specified a high risk of bias. This assessment was made based on the recommendations of the *Cochrane Handbook* [32]. We labelled uncertain or unknown risk of bias as ‘unclear’.

### Synthesis of data

Following the recommendation of *Cochrane Handbook*, to calculate the effect size, we used the mean change from baseline in the concentrations and SD of the variables of interest for both control and intervention groups. We determined the net changes in measurements (change scores) as ‘measure at the end of follow-up − measure at baseline’. We used the following formula to calculate standard deviation (SD) in situations where only the mean (SEM) was available: SD = SEM × square root (*n*), where *n* is the number of subjects. In situations where only median and range (or 95% confidence interval [CI]) were converted to estimate mean and SD values as explained before to estimate mean and SD values. When the outcome variable was available only in the graphic form, to digitalize and extract data, we used the software GetData Graph Digitizer 2.24 [33, 34]. Among eligible studies for any meta-analysis, heterogeneity exists in demographic characteristics of participants; further study designs might differ from one study to another. Ove overcome the issue of heterogeneity challenge, we used a random effects model (using the DerSimonian–Laird method) and the generic inverse variance method [35,36,37]. We evaluated the heterogeneity was using the I**^2^** index. *I* **^2^** values <50% corresponded to the use of fixed effect model, and the value of ≥50% linked with the use of fixed-effects and random-effects model. We expressed the effect sizes as difference between vitamin D intervention groups versus control group. We ran a sensitivity analysis using the leave-one-out method to evaluate the effect of each study on the overall effect size. A sensitivity analysis was conducted using the removes one study each time and repeats the analysis [38].

### Publication bias

We visually inspected the Begg’s funnel plot asymmetry, Begg’s rank correlation and Egger’s weighted regression tests to evaluate the potential publication bias. This step was followed by adjusting the analysis for the effects of publication bias using the Duval & Tweedie ‘trim and fill’ and ‘fail-safe N’ methods [39]. We used Meta-Analysis (CMA) V3 software (Biostat, NJ)[40] to conduct the meta-analysis.

## Results

### Summary of searches and study selection process

We identified a total of 213 unique citations from searches, of which, 187 records remained after removing duplicates. After screening the titles and abstracts, we found 33 articles eligible for further evaluation, of which, 21 were excluded for the following reasons: non-human studies, genetic or molecular studies (n = 8); reviews or editorial articles (n = 9); not enough data (n = 4); (see Figure 1). Therefore, we included 12 studies in the meta-analysis.

## Risk of bias assessment

There is an indistinct risk of bias in some of the items including allocation concealment, as well as participant and researcher blinding process. However, all evaluated studies have low risk of bias as stated by selective outcome reporting. Supplementary Table 2 presents the details of the quality of bias assessment.

### Characteristics of the included studies

Table 1 presents a summary of the characteristics of included studies. The eligible studies were published between 2007 and 2015 from four countries including the United States of America (four studies), United Kingdom (six studies), Italy (one study) and Hong Kong (one study). The number of participants included in studies ranged from 34 [11] to 114 [21]. Participants in two studies were only female [21,25]; while the proportion of women in other studies ranged from 14% [29] to 84.1% [27]. The mean age of participants ranged from 29 [22] to 67 [29] years. The duration range of follow-up across studies was from 8 weeks to 6 months. Studies used various vitamin D supplement doses. In eight studies cholecalciferol was provided in a dose ranging from 2500 IU/d [21] to 200,000 IU/d [28]. In three of the studies, ergocalciferol was used at a dose of 50,000 IU at baseline for 12 weeks [24] and a dose of 100,000 IU at baseline for 8 and 16 weeks [11,29]. In one study, paricalcitol was supplemented at a dose of 2 µg at baseline for 12 weeks [31].

## Pooled estimate of the effect of vitamin d supplement intake on FMD

**Figure 2.**
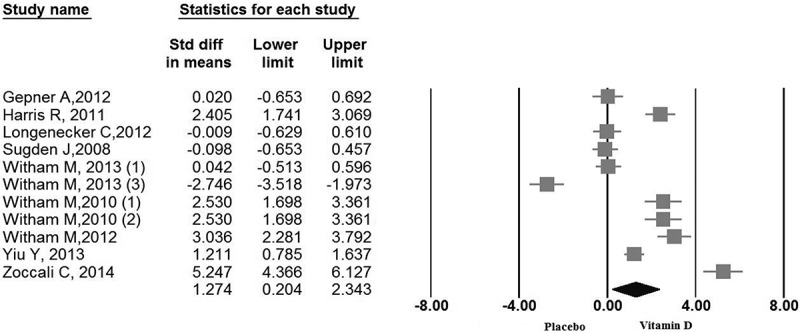
Forest plot displaying weighted mean difference and 95% confidence intervals for the impact of vitamin D supplementation on flow mediated dilation.

The pooled estimate (vitamin D intervention group versus control group) of the effect of vitamin D supplement consumption on FMD was 1.27 %, (95% CI 0.20 to 2.34, N = 11 arms, heterogeneity p = 0.054; I^2^ 51.2 %) across all studies (Figure 2).

### Sensitivity analysis

In leave-one-out sensitivity analyses, the pooled effect estimates remained similar for FMD, 1.27 %, (95% CI 0.20 to 2.34). This value indicates the constancy that the significant difference between the groups is the overall effect of all studies that were included in the meta-analysis.

### Publication bias

**Figure 3.**
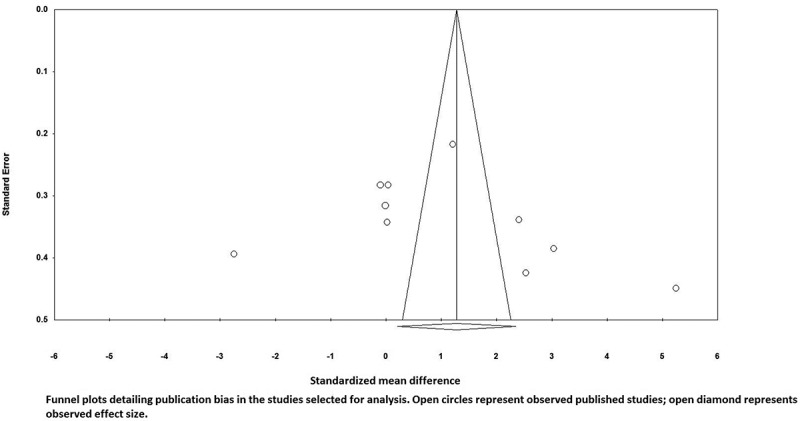
Funnel plots detailing publication bias in the studies selected for analysis flow mediated dilation. Open circles represent observed published studies; open diamonds represent observed effect size.

**Figure 4.**
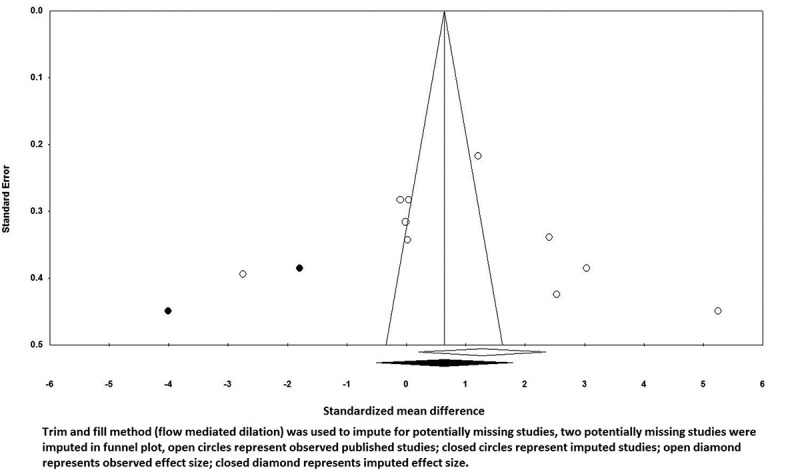
Trim and fill method (flow mediated dilation) was used to impute for potentially missing studies, two potentially missing studies were imputed in the funnel plot, open circles represent observed published studies; closed circles represent imputed studies; open diamond represents observed effect size; closed diamond represents imputed effect size.

A potential publication bias for the comparison of FMD levels between vitamin D supplemented groups and placebo groups was observed by visual inspection asymmetry in funnel plot (Figure 3). However, the presence of publication bias was not confirmed by Egger’s linear regression (intercept = 7.85, standard error = 7.42; 95% CI −8.94, 24.62, t = 1.05, df = 9.00, two-tailed P = 0.317) and Begg’s rank correlation test (Kendall’s tau with continuity correction = 0.37, z = 1.55, two-tailed P value = 0.119). After adjusting the effect size for potential publication bias, using the ‘trim and fill’ correction, two possibly missing studies were imputed in the funnel plot, hence some differences in effect size exist from the initial estimate (0.61%, 95% CI 0.43 to 0.80) (Figure 4). The ‘fail-safe N’ test indicates to bring the effect down to a non-significant (P > 0.05) value, 346 studies will be required.

## Discussion

In this study we have done systematic review and meta-analysis based on randomized controlled trial which investigated the role of vitamin D supplementation on endothelial function. The findings of this study suggested that vitamin D supplementation may improve vascular function. Our findings can be compared with previously published work. A double-blind, parallel group, placebo-controlled randomized trial, studied the effect of vitamin D supplementation in type 2 diabetic patients [11]. A single dose of 100,000 IU vitamin D_2_ (ergocalciferol) oral supplement versus placebo was investigated in 34 patients and showed an increase in FMD during 8 weeks of follow-up in subjects receiving vitamin D [11]. Tarcin et al. [12] examined the effects of vitamin D_3_ (300,000 IU) monthly for 3 months in vitamin D deficient however otherwise healthy adults and stated an increase in FMD levels versus control population. Furthermore, recent interventional studies demonstrated low vitamin D levels related to poor vascular health and improvement in brachial artery FMD, aortic stiffness, reactive hyperaemia index and blood pressure [41,42]. On the other hand, in a bigger follow-up trial with two different doses of vitamin D supplements (100,000 or 200,000 IU vitamin D_3_) vs placebo in 61 patients, at 8 and 16 weeks, no difference in FMD reported for the vitamin D groups [28]. The different vitamin D supplementation doses, means of administration and type of vitamin D supplements are some of the possible reasons that may be related to the different results in FMD among these previous studies. Although the direct route of the function is unknown; several mechanisms have been proposed by which vitamin D could improve endothelial function. Vitamin D receptors have been recognized in several cell types including vascular smooth muscle cells, endothelial cells and cardiac myocytes [4]. Vitamin D may possibly decrease proliferation of vascular smooth muscle, dysregulate systemic vascular calcium metabolism, decrease vascular resistance, downregulate proinflammatory cytokines, upregulate anti-inflammatory cytokines and reduces blood pressure by regulation of the renin–angiotensin system [43,44]. The synthesis of the active form of vitamin D by human endothelial cells may play at the local level to regulate the impacts of inflammatory cytokines on the vasculature [45]. However, findings of epidemiological investigations suggest that vitamin D supplementation has a positive effect on FMD and may reduce CVD risk.

Our study has some limitations. Firstly, consistent with other meta-analyses, the internal validity depended on the quality of individual eligible studies. Most of the studies included in our analyses had small sample sizes, possibly causing to overestimation of vitamin D supplementation effects on FMD. Trial with small sample sizes might be less robust, methodologically, and more susceptible to report larger effect sizes [46,47]. The number of appropriate studies was also rather small. Moreover, most of the studies were conducted in clinical population rather than general healthy population this is likely to affect the baseline levels of vitamin D and interested outcomes.

## Conclusion

This meta-analysis suggested that vitamin D supplementation may improve endothelial function, taking to account the limitations of the included studies. We suggest that more randomized controlled trials with larger sample sizes, more robust design and longer follow-up period should be considered for future investigations to provide clear answers to questions such is proper dose, the duration of supplementation and optimal vitamin D level, as well as potential mechanistic pathways.

## Supplementary Material

Supplementary Table 2

Supplementary Table 1
