# Supplementary material for: The impact of vitamin D supplement intake on vascular endothelial function; a systematic review and meta-analysis of randomized controlled trials
Source: Food Nutr Res. 2017 Mar 20;61(1):1273574. doi: 10.1080/16546628.2016.1273574 (PMC5404423; doi:10.1080/16546628.2016.1273574)
Supplement: Supplementary Table 2 [file zfnr_a_1273574_sm7800.docx]

**supplementary Table 2. Quality of bias assessment of the included studies according to the Cochrane guidelines.**

| **Studies** | **Random**  **sequence**  **generation** | **Allocation**  **concealment** | **Selective**  **reporting** | **Blinding of participants**  **and personnel** | **Blinding of outcome assessment** | **Incomplete**  **outcome data** | **Other**  **bias** |
| --- | --- | --- | --- | --- | --- | --- | --- |
| Gepner A, 2012([30](#_ENREF_30)) | L | L | L | L | L | L | L |
| Harris A, 2011([34](#_ENREF_34)) | L | L | L | L | L | L | L |
| Longenecker C, 2012([46](#_ENREF_46)) | L | L | L | L | L | L | L |
| Sokol S, 2012([36](#_ENREF_36)) | L | L | L | L | U | L | L |
| Sugden A, 2007([29](#_ENREF_29)) | L | U | L | L | L | L | L |
| Witham M, 2013([31](#_ENREF_31)) | L | L | L | L | L | L | L |
| Witham M, 2013([47](#_ENREF_47)) | L | L | L | L | L | L | L |
| Witham M, 2015([33](#_ENREF_33)) | L | L | H | L | L | L | L |
| Witham M, 2010([35](#_ENREF_35)) | L | L | L | L | L | L | L |
| Witham M, 2012([32](#_ENREF_32)) | L | L | L | L | H | L | L |
| Yiu Y, 2013([48](#_ENREF_48)) | L | L | L | L | L | L | L |
| Zoccali C, 2014([37](#_ENREF_37)) | L | L | L | L | L | L | L |

**L, low risk of bias; H, high risk of bias; U, unclear risk of bias.**
