# Supplementary material for: The impact of vitamin D supplement intake on vascular endothelial function; a systematic review and meta-analysis of randomized controlled trials
Source: Food Nutr Res. 2017 Mar 20;61(1):1273574. doi: 10.1080/16546628.2016.1273574 (PMC5404423; doi:10.1080/16546628.2016.1273574)
Supplement: Supplementary Table 1 [file zfnr_a_1273574_sm7755.docx]

| **Table supplementary 1. full Search terms and strategy used for systematically reviewing the articles** | | |
| --- | --- | --- |
| **No** | **Concept** | **Search terms** |
| **#1** | **Vitamin D** | Vitamin D[Text Word] OR vitamin d, 25-hydroxyvitamin D[Text Word] OR 25 hydroxyvitamin d[Text Word] OR cholecalciferol[Text Word] OR ergocalciferol[Text Word] OR 25-hydroxyvitamin D[Text Word] OR 25-hydroxy-vitamin D [Text Word] OR serum 25-hydroxyvitamin D[Text Word] OR serum 25-hydroxyvitamin D[Text Word] OR vitamin D2[Text Word] OR vitamin D3[Text Word] OR calcitriol[Text Word] OR calcidiol[Text Word]) OR calcifediol[Text Word] OR calciferol[Text Word] OR calciol[Text Word] OR calderol[Text Word] OR dihydrotachysterol[Text Word] OR dedrogyl[Text Word] OR dihydrotachysterol[Text Word] OR dihydroxycholecalciferol[Text Word] OR dihydroxyvitamin D[Text Word] OR dihydroxyvitamin D2[Text Word] OR dihydroxyvitamin D3[Text Word] OR doxercalciferol[Text Word] OR eldecalcitol[Text Word] OR ercalcidiol[Text Word] OR ergocalciferol*[Text Word] OR hidroferol[Text Word] OR hydroxycalciferol[Text Word] OR hydroxycolecalciferol[Text Word] OR hydroxycholecalciferol[Text Word] OR hydroxyergocalciferol*[Text Word] OR hydroxyvitamin D[Text Word] OR hydroxyvitamin D2[Text Word] OR hydroxyvitamin D3[Text Word] OR paricalcitol[Text Word] OR tachystin[Text Word] |
| **#2** | **Endothelial function** | "intima-media thickness"[Text Word] OR "carotid plaques"[Text Word]) OR "flow-mediated dilation"[Text Word] OR "nitrate-mediated dilation"[Text Word] OR "Flow-mediated dilatation"[Text Word]) OR "FMD"[Text Word]) OR "Nitric oxide"[Text Word] OR "Endothelial function"[Text Word] OR “vascular function”[Text Word] OR “IMT” [Text Word] |
| 3 | **Combination** | 1 AND 2 |
